# The Binding Mode to Orthosteric Sites and/or Exosites Underlies the Therapeutic Potential of Drugs Targeting Cannabinoid CB_2_ Receptors

**DOI:** 10.3389/fphar.2022.852631

**Published:** 2022-02-16

**Authors:** Rafael Franco, Paula Morales, Gemma Navarro, Nadine Jagerovic, Irene Reyes-Resina

**Affiliations:** ^1^ CiberNed. Network Center for Neurodegenerative Diseases, National Spanish Health Institute Carlos III, Madrid, Spain; ^2^ Molecular Neurobiology Laboratory, Department of Biochemistry and Molecular Biolomedicine, Universitat de Barcelona, Barcelona, Spain; ^3^ School of Chemistry, Universitat de Barcelona, Barcelona, Spain; ^4^ Medicinal Chemistry Institute, Spanish National Research Council, Madrid, Spain; ^5^ Department of Biochemistry and Physiology, Faculty of Pharmacy and Food Science, Universitat de Barcelona, Barcelona, Spain

**Keywords:** biased agonism, heteromer, health benefits, therapy, functional selectivity, cannabinoid receptor, CB2, allosterism

## Abstract

The classical terms agonists and antagonists for G protein coupled receptors (GPCRs) have often become misleading. Even the biased agonism concept does not describe all the possibilities already demonstrated for GPCRs. The cannabinoid CB_2_ receptor (CB_2_R) emerged as a promising target for a variety of diseases. Reasons for such huge potential are centered around the way drugs sit in the orthosteric and/or exosites of the receptor. On the one hand, a given drug in a specific CB_2_R conformation leads to a signaling cascade that differs qualitatively and/or quantitatively from that triggered by another drug. On the other hand, a given drug may lead to different signaling outputs in two different tissues (or cell contexts) in which the conformation of the receptor is affected by allosteric effects derived from interactions with other proteins or with membrane lipids. This highlights the pharmacological complexity of this receptor and the need to further unravel the binding mode of CB_2_R ligands in order to fine-tune signaling effects and therapeutic propositions.

## Introduction

G protein-coupled receptors (GPCRs) are the target of about 40% of current drugs (Hauser et al., 2017). Although the potential of GPCRs as therapeutic targets is still considered to be high, there have been only a few recent approvals of drugs targeting these receptors. The causes are multifactorial, but perhaps the main one is the increased demands, in terms of efficacy and safety, by regulatory bodies. Functional selectivity is a property of GPCRs that has recently become relevant to overcome the issues related to the lack of success of GPCR-targeted drug candidates (Chang and Bruchas, 2014; Franco et al., 2018). For therapeutic purposes, functional selectivity of a given compound acting on the targeted receptor could achieve the desired effect(s) while minimizing side effects. A simplified version of the full potential of functional selectivity is the concept of biased agonism. Biased agonism is now considered across all platforms developing therapeutic drugs in both industry and academia. A compound selectively modulating a signaling pathway could offer a suitable therapeutic benefit compared to a another agonist that could, in parallel, induce undesired signaling events. The structural features of the cannabinoid receptors (CBRs) offer more possibilities of biased signaling as the orthosteric site is not open to the extracellular milieu. Here we aim to review the multiple therapeutic possibilities resulting from targeting the cannabinoid receptor type 2 (CB_2_R) orthosteric and/or non-orthosteric sites. At present, CB_2_R appears as more promising in drug discovery than the cannabinoid receptor type 1 (CB_1_R) as some of CB_1_R agonists have psychotropic effects and an antagonist approved for human use (for weight control) was withdrawn due to serious side effects (Christensen et al., 2007; Sam et al., 2011). In fact, ligands for CB_2_R seem to be generally safe and irrespective of whether they are agonists or antagonists. Safety however will not be considered in the present article.

## Orthosteric and Non-Orthosteric Sites in the CB_2_R

### Modes of Ligand Binding to the Orthosteric Site

The canonical Gα protein subunit for CB_1_R and CB_2_R is Gαi. Therefore, activation of these receptors leads to inactivation of the adenylate cyclase with the subsequent decrease in cAMP and deactivation of protein kinase A-mediated signaling. However, activation of CBRs may also lead to activation of the mitogen-activated protein kinases (MAPK) signaling cascade, regulation of ion channels, and recruitment of ß-arrestins, with subsequent regulation of Tyr kinase activity among others (Alexander et al., 2021).

Binding to GPCRs using radiolabeled compounds leads to detect one or two sites. Two sites reflect two different populations that, in the well-studied adenosine A_1_ GPCR, correspond to the receptor uncoupled or coupled to the G protein. Uncoupled receptors display low affinity for agonists whereas G-protein coupled receptors display high affinity. These two affinity sites for the A_1_ receptor can only be detected using agonists, i.e. antagonists have similar affinities for G-protein coupled and uncoupled A_1_ receptors (see (Casadó et al., 1990) and references therein). To our knowledge radioligand binding to the CB_2_R results in the detection of one single population. The two radioligands frequently used for measuring the binding to cannabinoid receptors, [^3^H]WIN55,212-2 and [^3^H]CP 55,940, are considered very potent orthosteric agonists of both CBRs, CB_1_R and CB_2_R. Competition assays using radioligands and non-labeled compounds in heterologous cells expressing CB_2_R showed that affinities were consistent, i.e., WIN55,212-2 competed with similar low nanomolar affinity the binding of [^3^H]WIN55,212-2 and of [^3^H]CP 55,940. In similar conditions, a naturally occurring cannabinoid, cannabigerol, competed for the binding of [^3^H]WIN55,212-2 or [^3^H]CP 55,940 with a K_i_ in the micromolar range (Navarro et al., 2018b; Navarro et al., 2020b). This result did not fit with the decrease in cytosolic cAMP concentration obtained by nanomolar amounts of the compound. The main difference in the experimental setup was the use of isolated membranes for radioligand binding and of living cells for cAMP level measurements. The availability of novel approaches to obtain reliable receptor binding data in living cells is fortunately increasing, indeed, these methods do not require radiolabeled compounds. On using the SNAP-tag technology in cells expressing the tagged CB_2_R and a validated “hot” compound (Martinez-Pinilla et al., 2016), the K_i_ for cannabigerol competition was 152 nM (Navarro et al., 2018b; Navarro et al., 2020b). These results show that the measured affinity of a given compound depends on the probe used for binding and allows identification of different states of the receptor or different modes to accommodate the ligand within the orthosteric center. In the case of the CB_1_R, differences are more extreme as, in radioligand binding assays, natural cannabinoids may compete for the binding of [^3^H]WIN55,212-2 but not of [^3^H]CP 55,940. For instance, cannabigerol binding to CB_2_R is similar if measured using [^3^H]WIN55,212-2 or [^3^H]CP 55,940, whereas there is no significant competition of binding to the CB_1_R when [^3^H]CP 55,940 is used. In summary, cannabigerol binds to a subcompartment of the orthosteric site of the CB_1_R, i.e., the orthosteric site of this receptor may be simultaneously occupied by cannabigerol and [^3^H]CP 55,940. These relatively recent findings add useful information to understand the variety of actions that different cannabinoids exert and also the experimental diversity between laboratories in the values of affinity and potency. This diversity may also underlie the enormous potential of cannabinoid receptors to combat a wide variety of diseases (see (Franco et al., 2020) and references therein).

### Identification of Non-Orthosteric Sites

Cannabidiol, one of the main components of *Cannabis Sativa* L. has been instrumental to detect non-orthosteric centers in CBRs. This phytocannabinoid exerts physiological effects via a variety of receptors, located both in the cell surface and inside cells. Apart from interacting with CBRs, it may interact with serotonin and peroxisome proliferator-activated receptors (Banerjee et al., 1975; Russo et al., 2005; O’Sullivan et al., 2009; O’Sullivan and Kendall, 2010; Espejo-Porras et al., 2013; Fernández-Ruiz et al., 2013; De Gregorio et al., 2019; Franco et al., 2019b; Franco et al., 2020; Echeverry et al., 2021). At first cannabidiol was considered an orthosteric ligand able to partially activate cannabinoid receptors although with low potency (McPartland et al., 2007). Recent results in two different laboratories have shown that this compound can interact in an allosteric mode with the two CBRs (Laprairie et al., 2015; Martínez-Pinilla et al., 2017). For both receptors, CB_1_R and CB_2_R, it acts as a negative allosteric modulator (NAM) when co-administered with an orthosteric ligand. At CB_2_R it minimized the effects of JWH133 on the MAP kinase signaling pathway (Martínez-Pinilla et al., 2017). Thus, cannabidiol binds to an allosteric site at nanomolar concentrations while micromolar concentrations are required for significant binding to the orthosteric site. Accordingly, the *in vitro* results depend on the concentration while the *in vivo* actions at moderate doses should be mainly due to its binding to the allosteric site that has been very recently suggested to be close to the receptor entrance (Navarro et al., 2021) (See section: “Structural Insights into CB_2_R Binding Modes”). As would be expected from an allosteric mode of action, the binding of the compound to the allosteric site causes conformational changes in such a way that biases the effect of orthosteric agonists (Navarro et al., 2018a). A more recent report shows that structural changes in the molecule shifts negative to positive modulation (of the CB_2_R) thus confirming its allosteric nature (Navarro et al., 2021).

**FIGURE 1:**
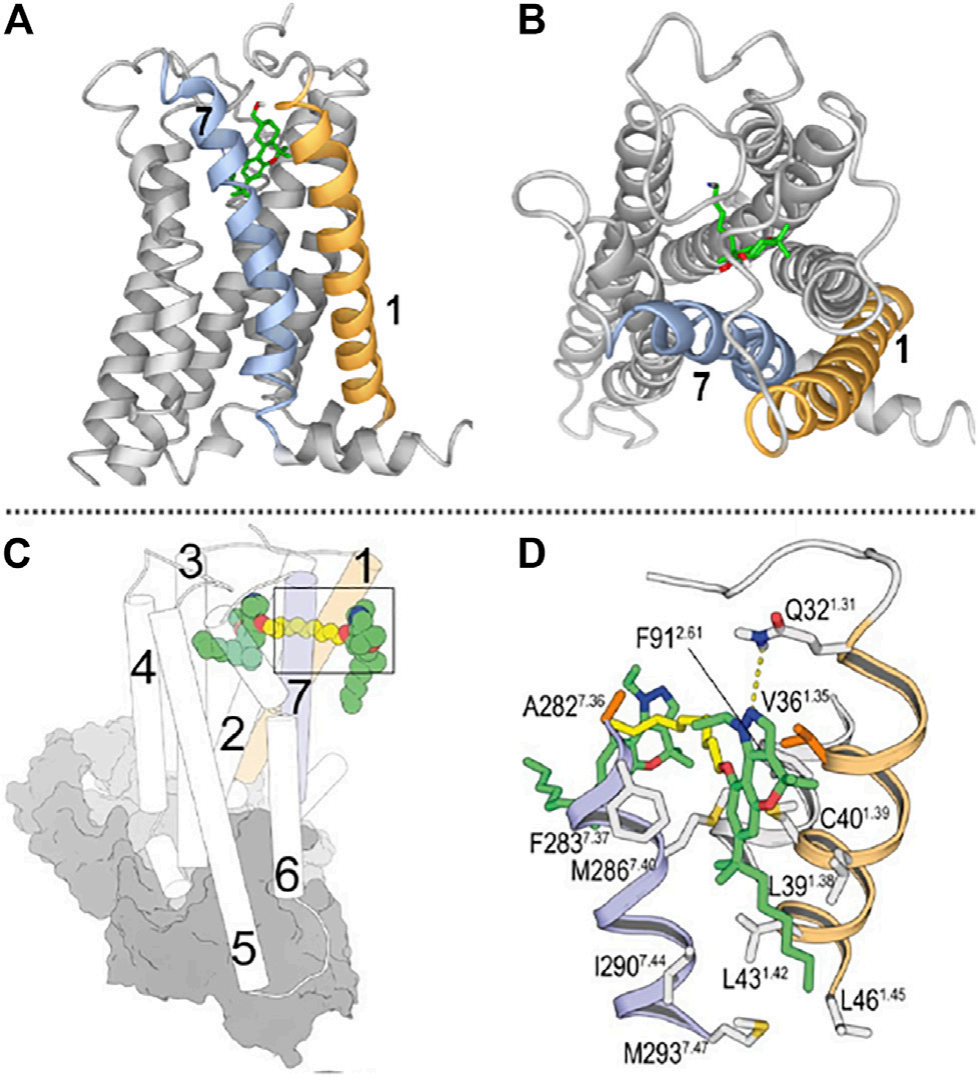
**(A)** Lateral view of the CB_2_R/AM12033 complex from the entrance portal formed by transmembrane helices (TMs) 1 and 7 (shown in orange and blue, respectively). **(B)** View from the outside of the cell of the CB_2_R in complex with the agonist AM12033 (PDB-ID 6KPF); ligand access from extracellular is blocked by the N-terminus and the EC loops. **(C)** General view of the binding mode of a CB_2_R bitopic ligand [molecule **22** in (Morales et al., 2020)] into the orthosteric site and the vestibule of the CB2R-Gi complex (depicted as cylinders for CB_2_R and grey surfaces for Gi). **(D)** Detailed view of the binding mode of ligand **22** into the receptor vestibule obtained during the MD simulations. TMs 1 and 7 are shown in orange and blue, respectively; and the pharmacophore units and spacer of bitopic ligands are shown in green and yellow tubes, respectively. **(C,D)** have been reproduced from our previously reported article (Morales et al., 2020); permitted reproduction under the terms and conditions of the Creative Commons Attribution (CC BY) license (https://creativecommons.org/licenses/by/4.0/).

Novel approaches to achieve signaling diversity and addressing success in drug discovery are attempting the design of bitopic ligands that bind the orthosteric site and an allosteric site (Lane et al., 2013; Mohr et al., 2013; Bradley and Tobin, 2016). By combining experimental and in silico approaches an allosteric site was identified at the entrance of the orthosteric binding site of the ß-adrenergic GPCRs (González et al., 2011). This site has been termed the -extracellular- vestibule (Dror et al., 2011) or entrance (Wang et al., 2013), also metastable (Fronik et al., 2017) or secondary (González et al., 2011) binding site. Exosite is also used to describe such non-orthosteric sites when they are located at the lipidic-receptor interface (Masureel et al., 2018). Bitopic ligands designed according to these findings improve subfamily selectivity (Medina et al., 2014; Masureel et al., 2018); they also offer signaling bias and better off-rates (Valant et al., 2012; Lane et al., 2013). Knowing that unlike GPCRs for polar compounds, CBRs do not have the orthosteric center accessible from the extracellular milieu, we designed bitopic ligands able to enter into the CB_2_R orthosteric site but also able to interact with amino acids located at the receptor transmembrane portals (Morales et al., 2020). Signaling assays in the CB_2_R wild-type and specific mutants led us to discover the first CB_2_R bitopic ligands. These compounds, which consist of two chromenopyrazole moieties linked by methylene spacers of different lengths, can bind to the orthosteric site and to an exosite. Bitopic ligands showed to be CB_2_R selective and, as depicted in Figure 1, may likely extend from the orthosteric site, the vestibule and an “allosteric exosite” able to accommodate the same moiety that sits in the orthosteric site.

## Structural Insights Into CB_2_R Binding Modes

As previously mentioned, in recent years, the CB_2_R has been resolved in its active (Hua et al., 2020; Xing et al., 2020) and inactive (Li et al., 2019) states, enlightening the structural knowledge of crucial domains for G protein activation as well as ligand binding. Not only CB_2_R but also CB_1_R and other class A lipid GPCRs have structural features that determine the lipophilic nature of their ligands (Hua et al., 2016; Hua et al., 2017; Krishna Kumar et al., 2019; Shao et al., 2019). On the one hand, the extracellular loops and the N-terminus of these receptors are generally structured over the orthosteric binding pocket occluding ligand entrance from the extracellular milieu. Moreover, transmembrane openings have been detected in these receptors acting as portals for lateral access of ligands to the binding crevice. Therefore, hydrophobic ligands such as phytocannabinoids need to diffuse through the lipid membrane to target binding sites. Figure 1A,B shows these features in the recently released structure of CB_2_R in complex with Gαi.

Class A GPCR allosteric sites are widely distributed in different receptor domains including intracellular, intrahelical or exosites. For instance, the CB_1_R has been resolved bound to the NAM ORG27569 and the agonist CP55940 (Shao et al., 2019). This crystal structure revealed the ability of ORG27569 to target an extrahelical exosite within the inner leaflet of the lipid bilayer. Even though few CB_2_R allosteric modulators have been reported and none resolved in complex with the receptor, molecular dynamic and mutagenic studies have recently shown the potential allosteric site of CBD in CB_2_R (Navarro et al., 2021). This report shows that CBD can bind to an allosteric cavity close to the receptor entrance in a transmembrane portal defined by transmembrane helices 1 and 7. As aforementioned, concomitant binding at orthosteric and allosteric/exosites has been shown at CB_2_R with chromenopyrazole bitopic ligands (Morales et al., 2020). Site-directed mutagenesis and molecular dynamic studies determined key interacting residues at transmembrane helices 1 and 7 which define the entry portal for these ligands (Figures 1C,D).

The CB_2_R structural understanding gained in the past few years will likely accelerate the rational drug design of CB_2_R modulators with optimal activity to address specific physiopathological conditions.

## Bidirectional Information Exchange Between Ligand and CB_2_Rs

On the one hand, functional selectivity can result from different agonists that activate different populations of receptors, but also from agonists that produce different conformational changes in the receptor that allow different qualitative and/or quantitative signaling outputs. On the other hand, a given agonist can give rise to different signaling outputs depending on the conformation of the receptor’s orthosteric site, which can vary depending on the cell type and the fate of the cell (Fuxe et al., 1998; Urban et al., 2007; Kenakin and Miller, 2010; Rajagopal et al., 2011; Fuxe et al., 2014; Ladarre et al., 2014; Navarro et al., 2020a; Franco et al., 2021).

By definition, allosterism produces conformational changes that alter the binding of agonists to the orthosteric site and, consequently, also modify (qualitatively or quantitatively) signal transduction. Important to highlight is that allosterism is bidirectional, i.e. an orthosteric compound binding to a receptor leads to conformational changes that likely alter the affinity of the binding of the allosteric compound to the receptor (Christopoulos and Kenakin, 2002; May and Christopoulos, 2003; Smith et al., 2011). In practice this means that if an allosteric compound is suspected on the basis of changes in affinity of radiolabeled compound to the orthosteric site, the orthosteric compound should modify the affinity of the binding of the allosteric compound to the allosteric site. In the field of GPCR, this requirement has made difficult the identification of allosteric compounds, as there are few to none radiolabeled compounds designed to measure binding to allosteric sites. In the case of CB_2_R, the discovery of bitopic ligands together with the structure of the receptor leaves no doubt about the possibility of regulating the functionality of the receptor by “touching” allosteric/exosites.

### Different Macromolecular Environments of the CB_2_R Impact agonist Binding and Effect

Can a given compound be more efficacious at targeting a cell that expresses CB_2_R in a particular conformation? and/or can a CB_2_R in a particular cell type be more likely to respond to the challenge of a given compound?

The pharmacology of cannabinoid receptors is complex. As discussed above, binding data can depend on the radioligand used as the probe, and the effects of a given compound on a given receptor are not always consistent across laboratories. At present we have enough data to realize that there are many possibilities for CB_2_R-mediated responses that may turn into novel and powerful possibilities for drug discovery.

**FIGURE 2:**
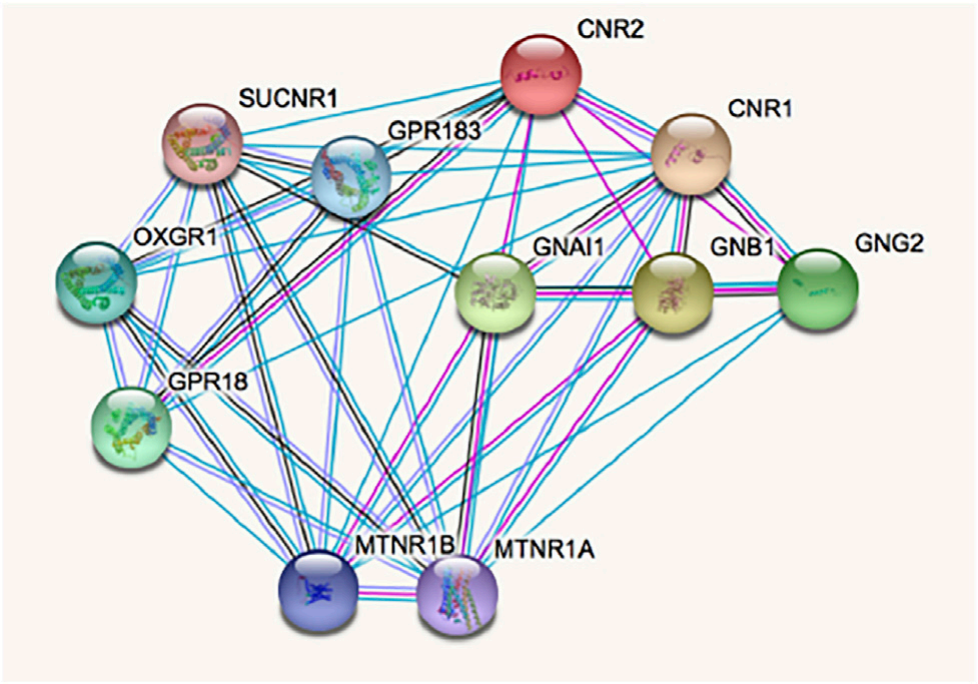
Interactions involving the CB_2_R according to STRING database for functional protein association networks. Abbreviations/gene products are: CNR2, CB_2_R; CNR1, CB_1_R; MTNR1A/1B, Melatonin GPCRs 1A/1B; OXGR1, Alpha-ketoglutarate receptor (a GPCR); SUCNR1, Succinate receptor 1 (a GPCR); GPR18 and GPR183 are orphan GPCRs; GNAl1, Guanine nucleotide-binding protein G(i) subunit alpha-1; GNB1, Guanine nucleotide-binding protein G(i) subunit ß-1; GNG2, Guanine nucleotide-binding protein G_i_/G_s_/G_o_ subunit gamma-2.

The complex pharmacology of the CB_2_R has likely delayed the identification of CB_2_R-containing macromolecular complexes, whose occurrence has been demonstrated in natural sources (i.e. not only in heterologous expression systems). Such interactions modify binding and/or function. Current data suggest that the receptor environment modifies the conformation and, accordingly, the binding and effects of orthosteric and non-orthosteric ligands. Interaction of the CB_2_R with other GPCRs may be searched in http://www.gpcr-hetnet.com/ (using the gene name: CNR2) (Borroto-Escuela et al., 2014). Figure 2 shows the STRING analysis of the interactions of the receptor which indicates mandatory interactions with G proteins, and interactions with the CB1R and with other GPCRs. In www.gpcr-hetnet.com and in Figure 2 interactions of CB_2_R with further GPCRs are not yet included (they have not yet been incorporated into the STRING database). Also missing are the recently described interactions of the CB_2_R with glutamate N-Methyl-D-Asp (NMDA) ionotropic receptors (Rivas-Santisteban et al., 2021). From a therapeutic perspective, the fact that CB_2_R may interact with other receptors that are also targeted by cannabinoids, for instance with GPR18 and GPR55, is of high interest (Balenga et al., 2014; Reyes-Resina et al., 2018; Martínez-Pinilla et al., 2019; Martínez-Pinilla et al., 2020; Rivas-Santisteban et al., 2021).

So far, no major change has been detected concerning the nature of the G protein coupling of CB_2_R in a macromolecular environment as it occurs for D_1_ and D_2_ dopamine receptors. Whereas the D_1_ is coupled to Gαs and D_2_ to Gαi, the macromolecular complex formed when the two receptors are co-expressed in the same neuron couples to Gαq (Rashid et al., 2007; Hasbi et al., 2009; George et al., 2014; Perreault et al., 2015). Notwithstanding, conformational changes that affect the binding and signaling outputs produced by a given agonist have been shown in the interactions with the Gαi-coupled CB_1_R (Callén et al., 2012; Sierra et al., 2015; Angelats et al., 2018), the Gαs-coupled adenosine A_2A_ receptor (Franco et al., 2019a), and the ionotropic NMDA receptor (Rivas-Santisteban et al., 2021).

In one of the first studies of biased agonism in GPCR heteromers (CB_1_R/CB_2_R), Navarro and co-workers showed that the allosteric effect of CBD was particularly noteworthy for the endocannabinoid anandamide but also that the effect tested using different agonists was smaller in the heteromer (Navarro et al., 2018a). These results confirmed that CBD acts as an allosteric modulator (for both receptors) also suggesting that the formation of the heteromer leads to conformational changes that make it less sensitive to the action of this phytocannabinoid. There are several examples of conformational changes induced by receptor-receptor interactions, i.e. by heteromer expression (Franco et al., 2007; Ferré et al., 2009; Franco et al., 2016). In the case of the CB_2_R, indirect evidence is provided by potentiation of receptor-mediated signaling when forming heteromers with the adenosine A_2A_ receptor (Franco et al., 2019a).

### Can Ligands Affect Conformation via Regulation of the CB_2_R Context?

The binding of orthosteric and non-orthosteric ligands alters the conformation of the receptor, but can ligands alter the environment? The answer to this question will take time as there is little background on the regulation of, for instance, heteromer formation.

Defining the target in the right context and delineating contextual changes due to ligand-induced regulation of the structure of the CB_2_R-contaning macromolecule, may further improve the rational design of therapeutic drugs (orthosteric and non-orthosteric) targeting the CB_2_R.
